# PRAGMATIC IMPLEMENTATION OF A BEHAVIORAL INTERVENTION PRE- AND POSTPANDEMIC: A TALE OF TWO TRIALS

**DOI:** 10.1093/geroni/igad104.1638

**Published:** 2023-12-21

**Authors:** Ellen McCreedy, Aleena Dewji, Laura Dionne, Vincent Mor, James Rudolph

**Affiliations:** Brown University School of Public Health, Providence, Rhode Island, United States; Brown University, School of public Health, Providence, Rhode Island, United States; Brown University School of Public Health, Providence, Rhode Island, United States; Brown University School of Public Health, Providence, Rhode Island, United States; Brown University, Providence, Rhode Island, United States

## Abstract

We describe two embedded pragmatic trials (ePCTs) of a personalized music intervention for nursing home (NH) residents with ADRD conducted pre-COVID (2019) and 2021 (COVID). Between trials, the implementation strategy was modified to incorporate learnings from the first trial and to address COVID-related restrictions. 54 intervention NHs (27 from Trial 1, 27 from Trial 2) comprised the sample. We compare the trials using the Framework for Implementation Fidelity (FIF) criteria: coverage (number of residents who received the intervention); duration (dose of music received); and frequency (how often nursing staff used the music with the residents). Adherence was worse in Trial 2 (post-COVID) versus Trial 1 (pre-COVID). On average, 12.7 (SD: 3.6) residents were exposed per NH in Trial 1 and 7.5 (SD: 5.5) in Trial 2. Median minutes of music per day was 27.1 (SD: 23.9) minutes in Trial 1 and 2.5 (SD: 5.2) minutes in Trial 2. A greater proportion of exposed residents had nursing staff administer music in Trial 1 (frequency; 0.4 (SD:0.3)) than Trial 2 (0.2 (0.3)). Despite changes to increase nursing engagement and clinical targeting, adherence to intervention was dramatically lower in Trial 2 than in Trial 1. While it is impossible to completely disentangle changes to the implementation strategy from the lingering effects of COVID, it is clear that the NH environment fundamentally changed during the pandemic. With that change comes the need to re-evaluate strategies for implementation and incentivizing participation

